# Rebelled epigenome: histone H3S10 phosphorylation and H3S10 kinases in cancer biology and therapy

**DOI:** 10.1186/s13148-020-00941-2

**Published:** 2020-10-14

**Authors:** Dorota Komar, Przemyslaw Juszczynski

**Affiliations:** Department of Experimental Hematology, Institute of Hematology and Transfusion Medicine, Gandhi 14 Str, 02-776 Warsaw, Poland

**Keywords:** Histone modifications, Kinases, H3, H3S10ph, Phosphorylation, Chromatin modifications, Cancer, Cancer therapy

## Abstract

**Background:**

With the discovery that more than half of human cancers harbor mutations in chromatin proteins, deregulation of epigenetic mechanisms has been recognized a hallmark of malignant transformation. Post-translational modifications (PTMs) of histone proteins, as main components of epigenetic regulatory machinery, are also broadly accepted as therapeutic target. Current “epigenetic” therapies target predominantly writers, erasers and readers of histone acetylation and (to a lesser extent) methylation, leaving other types of PTMs largely unexplored. One of them is the phosphorylation of serine 10 on histone H3 (H3S10ph).

**Main body:**

H3S10ph is emerging as an important player in the initiation and propagation of cancer, as it facilitates cellular malignant transformation and participates in fundamental cellular functions. In normal cells this histone mark dictates the hierarchy of additional histone modifications involved in the formation of protein binding scaffolds, transcriptional regulation, blocking repressive epigenetic information and shielding gene regions from heterochromatin spreading. During cell division, this mark is essential for chromosome condensation and segregation. It is also involved in the function of specific DNA–RNA hybrids, called R-loops, which modulate transcription and facilitate chromosomal instability. Increase in H3S10ph is observed in numerous cancer types and its abundance has been associated with inferior prognosis. Many H3S10-kinases, including MSK1/2, PIM1, CDK8 and AURORA kinases, have been long considered targets in cancer therapy. However, since these proteins also participate in other critical processes, including signal transduction, apoptotic signaling, metabolic fitness and transcription, their chromatin functions are often neglected.

**Conclusions:**

H3S10ph and enzymes responsible for deposition of this histone modification are important for chromatin activity and oncogenesis. Epigenetic-drugs targeting this axis of modifications, potentially in combination with conventional or targeted therapy, provide a promising angle in search for knowledge-driven therapeutic strategies in oncology.

## Background

Recent large-scale cancer genome projects like The Cancer Genome Atlas highlighted the role of epigenetic alterations in cancer development, as they revealed that about half of human cancers harbors mutations in chromatin-modifying proteins [1]. Post-translational modifications (PTMs) of histone proteins are main components of epigenetic regulatory machinery, as they determine the conformation of chromatin regions, regulate chromatin accessibility and operate as modulators of gene expression. Thus, appropriate abundance and hierarchy of histone proteins PTM are essential for normal development and maintenance of tissue-specific gene expression patterns. Disruption of this specific chromatin-context can lead to altered chromatin activity and disordered gene expression, hallmarks of malignant cellular transformation. Consistent with this, cancer cells typically exhibit aberrant landscapes of histone marks, and these differences are broadly accepted as therapeutic targets. Current “epigenetic” therapies directly or indirectly target predominantly writers, erasers and readers of histone acetylation and (to a lower extent) methylation, leaving other types of PTMs largely unexplored [2, 3]. Interestingly, histone-modifying enzymes were found to exhibit non-epigenetic functions, operating on substrates not directly associated with chromatin function; on the other hand, enzymes considered to catalyze reactions outside of chromatin (e.g., in signaling cascades), were found to modify histone proteins [4–6]. Such cross‐regulation of PTMs and signaling pathways appears a fundamental mechanism for regulation of gene expression. Thus, both intra- and extranuclear effects of the inhibition of enzymes catalyzing those modification should be taken into consideration when evaluating new signal transduction inhibitors and epigenetic drugs. In this view, histone phosphorylation appears to be especially interesting, as most of histone kinases were historically characterized merely as transducers in signaling pathways, and their role in cancer is still being investigated predominantly in this aspect, despite growing evidence of their chromatin-connected function.

Phosphorylation of serine 10 on histone H3 (H3S10ph) is an unique histone modification as it is involved in two structurally opposed processes: transcriptional activation and chromatin relaxation, or chromosome compaction during cell division [7–14]. This suggests, that H3S10ph, rather than directly affecting chromatin structure, acts indirectly, serving as a protein binding platform, or a chromatin signpost, and the effect of H3S10ph on its neighborhood as well as its role, demand context-dependent interpretation. As histone H3S10 phosphorylation is involved in proliferation and transcriptional activation, it is not surprising that this epigenetic mark emerged as an important player in the initiation and propagation of cancer [15–18]. In addition, increase in H3S10ph was observed in and associated with poor prognosis in several cancers, including invasive breast cancer [19, 20] esophageal squamous cell carcinoma [21], gastric cancer [22, 23], glioblastoma [24], melanoma [25, 26], nasopharyngeal carcinoma [18], and others.

In this review, we address the role of H3S10 phosphorylation in chromatin biology. We present multiple H3S10 kinases that are known for their extra-chromatin functions; for example, in signaling cascades typically activated in cancer, we discuss their role in cancer and their inhibition as a potential therapeutic strategy.

## H3S10 phosphorylation in chromatin dynamics and cell cycle regulation

In contrast to other modifications of H3 tail like H3K9me2 or H3K9ac, H3S10ph mark does not become erased as cell enters mitosis [27], but its abundance increases during cell division, compared to the interphase [28, 29]. High H3S10ph occupancy along the entire length of chromosomal arms is typically observed in prophase and metaphase. In anaphase, the H3S10ph is significantly decreased and is limited to the chromatid tips [30]. Since highly condensed metaphase chromosomes are heavily H3S10 phosphorylated in most organisms analyzed thus far, increased H3S10ph was proposed a distinctive mark for dividing cells [11, 31]. This epigenetic modification, however, is not solely a marker of mitotic progression, but plays an important role in chromosome dynamics during cell division. In early studies, overexpression of H3 mutant, in which S10 is replaced by non-phosphorylable alanine led to abnormal chromosome segregation, resulting in extensive chromosome loss during mitosis, demonstrating that H3 serine 10 phosphorylation is required for proper chromosome dynamics [32–34]. However, in contrast to some other histone marks associated with chromatin compaction, in vitro studies revealed that H3S10ph does not induce spontaneous chromatin condensation and formation of higher order structures [35], suggesting that chromosomal condensation is mediated by other mechanisms, possibly involving additional proteins interacting with H3S10ph. Indeed, H3S10ph mediates large-scale exclusion of heterochromatin protein 1 (HP1) from chromosome arms in mitosis [10]. Conversely, disruption of H3S10ph by expression of non-phosphorylable H3S10 alanine mutant results in ectopic spreading of a heterochromatin-associated H3K9me2 into adjacent euchromatic regions, resembling the phenotype observed in Drosophila Jil-1 kinase mutants [28]. As H3K9me-binding HP1 tethers a chromosomal passenger complex, a key regulatory factor that controls chromosome segregation during mitosis, to the centromeric heterochromatin, it was speculated that this H3S10ph-mediated HP1 exclusion allows redirection of chromosomal passenger complex to mitotic centromeres [10, 36]. In line with this model, deregulation of H3S10ph affected kinetochore function and chromosome segregation [37, 38].

In addition to displacement of HP1, H3S10ph can affect interactions of chromatin also with other proteins, regulating mitotic chromosome dynamics. For example, in *S. cerevisiae*, H3S10ph is required for recruitment of the histone deacetylase HST2, which mediates deacetylation of H4K16, freeing the H4 tail to interact with the surface of neighboring nucleosomes and promoting fiber condensation [39]. Another mechanistic model proposes that H3S10ph shapes chromatin architecture, promoting detachment of the chromatin from nuclear scaffold [11]. Consistent with this model, H3S10ph mark overlaps with early replicating genes that are relocated to active transcription factories in the inner part of nuclei prior to transcription [40], but is absent in lamina-associated domains, proximal to nuclear envelope [28]. Moreover, in cells that lack Repo-Man (CDCA2), a PP1 (protein phosphatase 1) guide subunit that targets PP1 to chromosomes to dephosphorylate H3S10, chromosomes relocate from outer regions to the middle of nuclei [41]. H3S10ph-mediated nuclear positioning would be then facilitating both transcription and access to the replication machinery during interphase, and chromosome condensation during mitosis [28].

**Fig. 1:**
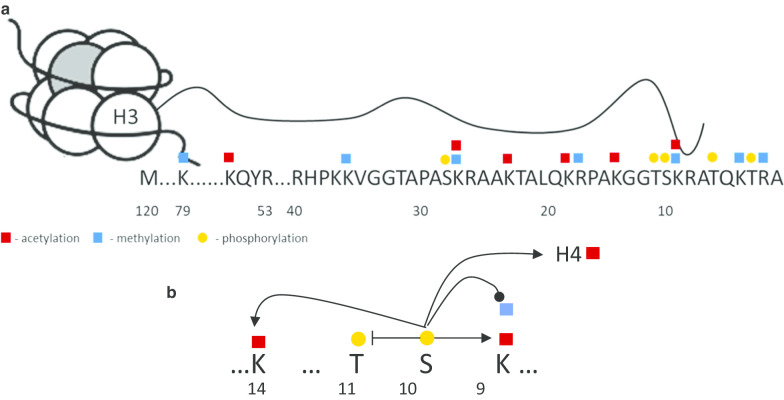
**a** Common posttranslational modification of histone H3 tail. Target residues for acetylation, methylation and phosphorylation in N-terminal histone region are shown. **b** Crosstalk between H3S10 phosphorylation and other histone post-translational modifications. In *cis* H3S10ph: (1) modifies binding of histone writers and inhibits phosphorylation of neighboring T11 [76]; (2) modulates epigenetic information coming from K9 methylation by regulating K9 methyltransferases [10, 56, 67–69] and demethylases [74, 75]; (3) as K9 can be both methylated and acetylated, H3S10ph also affects K9ac, it acts in synergy with this histone mark and increases efficiency of acetylation reactions [57, 105]; (4) same effects as for K9ac can be seen for K14ac [7]. H3S10ph can also affect histone post-translational modifications in *trans*, like histone H4 acetylation, attracting H4 acetyltransferases and protecting from deacetylase action [62, 65]

Interestingly, H3S10ph is not uniformly inherited through the cell cycle. It is predominantly associated with an “old” H3 at early mitosis and appears in “new” H3 with the progression of mitosis reaching symmetrical distribution [42]. Inhibition of the Aurora kinase (AURK) B, one of the main H3 mitotic kinases, does not change this pattern, despite significant reduction in H3S10ph levels, but does affect distribution of K9me2 on the new H3. This strict control of H3S10ph deposition reaffirms its importance during mitotic events, highlighting the crosstalk between H3S10ph and other histone marks, and suggests a broader role for H3S10ph in histone bookmarking and epigenetic memory (Fig. 1).

## H3S10 phosphorylation, genomic integrity and DNA damage response

Recent studies reveal a new role of H3S10ph mark, linking its deposition and chromatin structure to genome integrity. H3S10ph mark is abundantly deposited within actively transcribed regions forming hybrid structures composed of a nascent RNA, DNA template and a displaced nontemplate single-stranded DNA, called R-loops [43, 44]. R-loops are important regulatory intermediates that can stimulate or inhibit transcription, either by blocking promoter DNA methylation and RNA polymerase pausing, or by promoting transcriptional termination [45, 46]. In addition to the regulatory functions in gene expressions, R-loops formation can lead to DNA damage. As forming R-loops expose single DNA strand, they make it vulnerable to endonucleases and other enzymes causing DNA damage. In addition, R-loops hamper progression of replication forks, promoting DNA breaks at the collision sites [47]. Interestingly, R loop formation in activated B lymphocytes facilitates class switch recombination of immunoglobulin genes [48]. Since R-loops can also be formed at the MYC locus, it was proposed that they facilitate chromosomal translocations bringing MYC oncogene under control of Ig enhancers in certain B-cell malignancies [49, 50].

Since R-loops are a potential threat to genomic integrity, it is particularly interesting that their formation is associated with certain chromatin modifications, including H3S10 phosphorylation. H3S10ph occurs specifically at least at a subset of R-loop-forming regions. Overexpression of RNAse H, resolving R-loops, abrogates this effect, indicating that H3S10 phosphorylation is a specific cellular response to R-loops. H3S10ph at these R-loops leads to chromatin compaction, and dysregulation of H3S10 phosphorylation leads to increased genomic instability [43]. Histone mutants unable to phosphorylate H3S10 suppress the R-loop associated genomic instability, indicating that H3S10 is required for deleterious consequences of R-loops, and, perhaps, that H3S10 phosphorylation separates regulatory R-loops from those associated with DNA-damage [51]. However, mechanistic insights into H3S10ph consequences for chromatin dynamics at DNA damage—prone R-loops is lacking; likewise, specific kinase(s) responsible for H3S10 phosphorylation at R-loop forming regions has/have not been yet identified. As premature chromatin condensation causes chromosome fragility [52], H3S10ph-induced chromatin compaction might be a potential explanation to these effects. However, as H3S10-induced chromatin compaction is cell cycle phase-specific and mediated by mitotic kinases AURKA/B, this hypothesis needs further mechanistic studies.

The deleterious role of H3S10 at the sites of DNA damage has been also suggested by the studies by Sharma [53]. Ionizing radiation-induced DNA damage led to reversible loss of H3S10ph specifically in G1-phase of cell cycle, mediated by MKP1 phosphatase recruited to damage foci. Intriguingly, blocking of H3S10 dephosphorylation by MKP1 inhibition impaired DNA repair process and resulted in cell death. These data suggest that H3S10ph favors genomic instability by hampering DNA damage response in G1 phase of cell cycle, also raising the possibility of combinatorial modulation of H3S10ph with specific inhibitors to increase the sensitivity of cancer cells in G1-phase of cell cycle to irradiation.

## H3S10 phosphorylation in the regulation of transcription in interphase

Interphase chromatin is broadly occupied by H3S10ph, especially in gene-rich regions. H3S10 phosphorylation is abundant at domains of early DNA replication timing and correlates negatively with H3K9me2 and lamina-associated domains, suggesting that H3S10ph plays a role in activation of transcription [28]. Indeed, phosphorylation of specific residues of histone H3 at promoters of proto-oncogenes, *c-FOS* and *c-JUN*, resulted in their rapid transcriptional induction. Interestingly, the same regions were reported to be especially susceptible to lysine hyperacetylation, suggesting a link between H3 phosphorylation and acetylation. In fact, a more detailed analysis showed that histone H3 on *c-FOS*- and *c-JUN*-associated nucleosomes becomes doubly-modified, the same H3 tails becoming both phosphorylated and acetylated upon gene activation [54]. Dual H3S10ph-K14ac histone mark was soon associated with many active promoters [7, 55, 56].

In vitro pull-down assays with N-terminal tail of histone H3 phosphorylated at S10 followed by mass spectrometry identified 6 members of 14–3–3 protein family as H3S10ph binding partners [57, 58]. 14-3-3s form a well-conserved, abundant family of phosphoacetyl-binding proteins that can interact with over 300 partners including many transcriptional regulators and chromatin-modifying proteins (reviewed in [59]). Acetylation of histone’s H3 lysines 9 and 14 increases 14-3-3 affinity to H3S10ph [56, 60]. Binding of 14-3-3 proteins to H3S10ph-K14ac, protects it from dephosphorylation and stabilizes gene expression [61]. These data confirm strong, gene activation-promoting function of simultaneous H3S10 phosphorylation and H3 acetylation. Moreover, phosphorylation of H3S10 induces also other subsequent modifications leading to transcription-facilitating chromatin topography. For example, proto-oncogene serine/threonine-protein kinase PIM1-mediated S10 phosphorylation of H3 at the genomic region of FOSL1 already enriched with K9 acetylation attracts 14-3-3s that recruit histone acetyltransferase MOF. MOF deposits H4K16ac, a histone modification that interacts with bromodomain-containing protein 4 (BRD4), which subsequently recruits positive transcription elongation factor, P-TEFb, phosphorylating RNA pol II carboxy terminal domain (CTD) at S2. This complex sequence of chromatin modification events leads to the release of RNA pol II from the promoter proximal pause and transcriptional elongation of the *FOSL1* gene [62, 63]. H3S10 phosphorylation at certain promoters also leads to chromatin remodeling, giving transcription factors access to regulatory DNA sequences. Crucial role in this process is also played by 14-3-3 phosphoacetyl adapter, complexing with MSK1 and BRG1, the ATPase subunit of the SWI/SNF remodeler [64]. In addition, H3S10ph protects acetylated histone H4 from deacetylation. In unstressed human cells, H3S10ph stabilizes H4K5/K8 acetylation. Upon stress, phosphatase PP1 dephosphorylates H3S10, what allows deacetylation of histone H4 by histone deacetylases HDAC1, 2 and 3. Deacetylation of H4 destroys binding platform for BRD4, what causes BRD4 detachment and enables transcriptional elongation of stress-induced genes [65].

Lysine K9 in H3 lies in the immediate neighborhood to S10. K9 carries not only acetylation mark, recruiting factors associated with transcriptional activation, but also methylation mark, which binds factors associated with transcriptional repression [66]. This susceptibility of K9 to distinct modification led to the hypothesis that histone phosphorylation may be a component of a switch mechanism between gene silencing and activation. Indeed, soon a “phospho/methyl switch” mechanism that causes dissociation of a reader of the lysine methylation mark and loss of “repressive character” of chromatin region has been observed. Presence of H3S10ph (and H3K14ac) adjacent to H3K9 methylation affects the binding of HP1 and causes its release from H3K9me-decorated silenced chromatin [10, 56, 67–69]. Additionally, H3S10ph prevents deposition of H3K9me2, and exchange of H3S10ph with non-phosphorylable H3.3S10A results in ectopic spreading of H3K9me2 into adjacent euchromatic regions. Interestingly, this antagonism is reciprocal, as in H3K9 methyltransferase mutant, H3S10ph domains expand, revealing that H3S10ph deposition is restricted by H3K9me2 [28, 70]. In agreement with this finding, a phosphatase complex Repoman1/PP1 that dephosphorylates H3S10 and H3S28 [71], regulates the formation of heterochromatin and is necessary and sufficient for HP1 recruitment and repressive histone modifications [41]. Also in mouse embryo model, H3S10ph limited the expansion of heterochromatin features: H3K9me2 and DNA methylation [72]. Furthermore, H3S10ph positively affects H3K4 methylation by making histone H3 a more attractive substrate for a SET domain of KTM2A/MLL1 complex and enables this methyltransferase complex to outcompete HP1- and Polycomb-mediated chromatin condensation [73], indicating that safeguarding nucleosomes from heterochromatin marks leads to reinforcement of factors associated with active transcription.

It is important to mention that not all readers of methylated K9 are sensitive to H3S10 phosphorylation. Likewise, S10ph does not always antagonize heterochromatin formation/spreading. For example, demethylase Jumonji-containing-protein 2A (JMJD2A) is unable to demethylate H3K9me3 if H3S10 is phosphorylated [74, 75]. In this context, H3S10ph shields H3K9me3 from the demethylase and helps to preserve heterochromatin configuration. Moreover, H3S10ph controls the access of writers/erasers of other histone marks, beside K9 methylation, like H3T11 that cannot be phosphorylated by recombinant Chk1 kinase in the vicinity of S10ph [76].

Of note, even though H3S10ph facilitates transcription, it is not a prerequisite for the transcription to occur. In stress-induced system in *Drosophila melanogaster*, H3S10ph was not upregulated in the genes activated in response to the heat shock, nor was it needed for the transcription of those genes in H3S10 kinase mutant [77, 78]. These findings indicate that H3S10ph affects chromatin structure/function both directly and indirectly, by modifying the composition of chromatin binding protein platforms. Thus, interpretation of H3S10 functions must be carried cautiously, in the context-dependent manner.

Taken together, H3S10 phosphorylation regulates transcription by: (1) promoting recruitment of chromatin proteins that support active transcription; (2) shielding gene regions from heterochromatin factors; (3) dictating the hierarchy and landscape of subsequent histone post-translational modifications; (4) contributing to the formation of binding platform for effector proteins.

## Mitotic H3S10 kinases

Deposition of H3S10ph mark during mitosis is mediated by members of the Aurora kinase family, comprising three highly homologous serine–threonine kinases which expression and activity peaks in M phase (Table 1). Aurora kinase A (AURKA) has an essential role during cell division, being involved in centrosome maturation and separation, spindle assembly, bipolar spindle microtubule formation, microtubule nucleation, cytokinesis, and mitotic entry and exit (reviewed in [79]). Consistent with its function, AURKA localizes to the centrosome (and is often referred to as a centrosomal kinase) and along microtubule-based mitotic spindle [80]. AURKA phosphorylates multiple substrates that facilitate cell cycle progression and are involved in maintenance of genome integrity, including H3S10, and its expression correlates with the deposition of this mark [81]. AURKA homolog, AURKB, localizes to the kinetochores and the anaphase central spindle as part of the chromosomal passenger complex [82]. It phosphorylates H3S10 during mitosis to aid chromosome condensation [83]. Overexpression of AURKA and AURKB has been noted in many human cancers, where these proteins exhibit oncogenic properties [84–87]. Consistent with this, Aurora kinases appear as rational therapeutic targets and many inhibitors are currently at different stages of preclinical and clinical development (Table 2). Preliminary results of clinical studies indicate that at least some of these inhibitors bring clinical benefits, such as synergy with conventional chemotherapy, improved time to disease progression, prolonged progression-free survival and the duration of disease stabilization [79, 88, 89]. In particular, these beneficial effects were observed in highly aggressive tumors with high mitotic index, such as high-risk acute myeloid leukemia [90]. Importantly, although Aurora kinases exhibit pleiotropic activity and utilize a broad range of substrates, at least a part of clinical activity of their inhibitors can be ascribed to alterations in H3S10ph abundance. For example, downregulation of AURKA is required for leukemia cell differentiation, and resulting loss in H3S10ph enables recruitment of methyltransferase G9a to target gene promoter and spreading of H3K9 methylation and decrease in expression of cell division factors including CDCA7 and CDC25A. Similar effects were observed after AURKA inhibition with alisertib [91]. Exposure of an acute myeloid leukemia cell line THP-1 to alisertib induces cell differentiation into monocytes [92]. In gastric cancer, AURKB controls cell cycle through H3S10ph-dependent regulation of the *CCND1* gene promoter activity [93]. Also genetic instability caused by the deregulation of Aurora kinases is associated with H3S10ph. Phosphorylation of H3S10 by AURKB is required to initiate chromosome condensation [14] but increased mitotic phosphorylation of histone H3 caused by AURKB overexpression leads to chromosome lagging and aneuploidy after cell division [94]. On the other hand, also AURKA/B inhibition decreases the global abundance of mitotic H3S10ph and leads to aberrant mitoses [95]. AURKA inhibition-associated genomic instability might be synthetically lethal in cancers with defects in certain DNA repair genes. For example, breast cancers with mutations in *BRCA2* are more sensitive to AURKA inhibitors [96].

**Table 1:** H3S10 kinases and their H3S10ph-related function in oncogenesis

Kinase	H3S10-specific consequences of activity in oncogenesis and in cancer cells
AURKA	Role in mitotic chromosome condensation. Inhibition facilitates leukemia cell differentiation [91, 92]
AURKB	Role in mitotic division [142]
IKKα	NF-κB, inflammation, immune-response [129–131, 143]
JNK	Certain oncogenes activation through H3S10ph in their promoters in response to oncogenic stimuli [98, 99, 110]
AKT1	Potential role in gene expression activation in response to oncogenic compounds together with RSK2 [144]
MAP3K8	Activation of IEG/oncogene-FOS and cellular transformation in response to DNA damage [145]
CDK8	Activation of IEGs/oncogenes [111, 112]; downregulation of superenhancer-dependent genes in acute myeloid leukemia [118]
MSK1/2	Activation of IEGs/oncogenes FOS and JUN [7, 12]; response to carcinogens [16, 64, 100]; inflammation [134, 135]
PIM1	Activation of proto-oncogene FOSL1 [63]; oncogenic miR-17-92 [146] cooperation with MYC to induce cellular transformation [62, 63]; potential role in mitotic checkpoint regulation [147, 148]

**Table 2:** Small molecules shown to modulate H3S10ph deposition, FDA-approved for clinical trials

Name	Target	Clinical trial no	Phase	Disease	H3S10ph source
Barasertib (AZD1152-HQPA|AZD281)	AURKB/A	NCT03366675; NCT03217838	Phase 1/2	Small cell lung cancer, acute myeloid leukemia /high-risk myelodysplastic syndrome, advanced solid tumors	[149]
Flavopiridol	CDKs, including CDK8	NCT03593915; NCT03441555; NCT03563560	Phase 1/2	Myelodysplastic syndromes; acute myeloid leukemia	[150, 151]
BI-847325	AURKA/B/C	NCT01324830	Phase 1	Solid tumors	[152]
Alisertib	AURKA	NCT02860000	Phase 2	Breast cancer	[91]
SEL120	CDK8	NCT04021368	Phase 1	Acute myeloid leukemia or high-risk myelodysplastic syndrome	[153] and unpublished observations
SEL24/MEN1703	PIM kinases	NCT03008187	Phase 1/2	Acute myeloid leukemia	[154]
PIM447	PIM kinases	NCT02370706	Phase 1	Myelofibrosis	[62, 63]

## Interphase kinases

### Mitogen- and stress-activated kinases 1 and 2 (MSK1/2)

Interphase phospho-H3S10 mark is deposited on actively transcribed genes by multiple kinases (Table 1), activated in response to various extracellular stimuli. Thus, H3S10 mark is a dynamic modification coupling extracellular signals to transcriptional responses and specific changes in cell physiology. Canonical mechanism of inducible H3S10 deposition involves mitogen-activated protein kinase (MAPK) pathway, activated by growth factors, mitogens, serum response, cytokines and cellular stressors. Operating downstream of the ERK1/2 and p38 MAPKs, MSK1 and 2, orthologs of Drosophila Jil-1 kinase, the canonical H3S10 kinase in the fruit fly, induce rapid H3S10 phosphorylation at the promoter and enhancer regions of immediate early genes (IEGs) [7, 12]. IEGs are identified by their rapid and transient transcriptional induction, requiring no new protein synthesis. Multiple IEGs encode transcription factors, such as *c-MYC, c-FOS, c-JUN, FOS1L, EGR1*, that are responsible for transcriptional reprogramming of cells exposed to environmental stress-inducing stimuli. Since many of these IEGs are oncogenes, and H3S10 phosphorylation is typically triggered by factors known to be potentially carcinogenic, such as exposure to 12-O-tetradecanoylphorbol-13-acetate (TPA) [64, 97], formaldehyde [98], cigarette smoke [99], ionizing radiation [53], H3S10ph may represent a mechanistic link between carcinogens and cellular transformation. This hypothesis is supported by the studies indicating that TPA or epidermal growth factor (EGF)-induced MSK1 activity and H3S10ph is required for transformation of mouse epidermal cells [16, 100]. Consistent with these observations, MSK1/2 knockout mice treated with the carcinogen 7,12-dimethylbenz[a] anthracene develop significantly fewer skin tumors than their wild type littermates [101]. An increase in MSK1 activity and the steady-state levels of phosphorylated H3S10 is also observed in h-Ras transformed mouse fibroblasts, Ciras-3. These Ras-transformed cells exhibited increased steady-state levels of the IEG products c-JUN, c-FOS and COX-2. Furthermore, MSK1 knockdown in Ciras-3 cells limits anchorage-independent growth, a hallmark of cellular transformation [102]. H3S10ph levels are also increased in breast cancer cells and, similarly to other epithelial cancers, increase after TPA treatment via the activity of RAS/MAPK pathway. Promoter region of a breast cancer marker gene and an inferior prognostic factor, trefoil factor 1 (TFF1) is occupied by MSK1 and decorated with H3S10ph [103]. The MSK-mediated phosphorylation of H3S10 leads to the recruitment of 14-3-3 adaptor and BRG1 to the enhancer and upstream promoter regions of TFF1 [104]. Subsequently, chromatin remodeling at the TFF1 regulatory elements leads to the RNA polymerase II CTD phosphorylation, initiating TFF1 expression. Taken together, MSK1 is required for carcinogen/tumor promoter-induced cell transformation through phosphorylation of histone H3 at Ser10 and transcriptional activation of oncogenes [16, 99, 100].

### Ribosomal S6 kinase 2 (RSK2)

RSK2, another MAPK-pathway kinase regulated downstream of ERK1/2, plays similar function to MSKs as it is able to phosphorylate H3S10, albeit with fourfold lower activity for this substrate [105]. Stimulation of mouse sarcoma C3H10T1/2 cells with EGF that causes IEG expression and deposition of activating H3S10ph and H3K14ac histone mark is correlated with RSK2 activation [7]. Also in mouse skin epidermal cells line JB6 Cl41, phosphorylation of RSK2 was increased within 5 min after TPA or EGF stimulation [106]. Cell proliferation and colony formation was significantly enhanced in cells with stable RSK2 expression in comparison to RSK2^−/−^ mouse embryonic fibroblasts. These results show that RSK2 plays an important role in cellular transformation induced by tumor promoters such as EGF and TPA [106]. Importantly, RSK2-mediated histone H3 serine 10 phosphorylation is confirmed by using RSK2-deficient Coffin-Lowry syndrome (CLS) patient cells. Fibroblasts derived from a CLS patient that possess RSK2 mutation failed to exhibit EGF-stimulated phosphorylation of H3, although H3 was phosphorylated during mitosis. Re-introduction of the wild-type RSK2 to RSK2 deficient CLS patient cells restores EGF-induced histone H3 phosphorylation at serine 10 [107]. Interestingly, RSK2-mediated p53 phosphorylation in the cytoplasm induces nuclear p53 accumulation and increases interaction between RSK2 and histone H3, promoting S10 phosphorylation in response to UV or EGF-treatment, while p53 deficiency diminishes RSK2-mediated H3S10ph [108]. In addition, another MAP kinase, c-Jun NH(2)-terminal kinase (JNK) was shown to be involved in H3S10 phosphorylation [109]. JNK-mediated H3S10 phosphorylation was observed after stimulation with carcinogens like cigarette smoke [99], formaldehyde [98] and arsenite [110].

### Cyclin-dependent kinase 8 (CDK8)

IEGs seem to be especially dependent on H3S10 regulation, as another H3S10 kinase, CDK8 was also identified as a positive regulator of IEGs. CDK8 is a part of mediator complex with a role in gene-specific regulation of transcription, thus functioning as a bridge between basal transcriptional machinery and transcription factors targeting specific genes. During serum-stimulation CDK8 is required for the proper transcription of IEGs, including oncogenic transcription factors [111]. CDK8-dependent H3S10 phosphorylation stimulates H3K14 acetylation by the GCN5L acetyltransferase, associating with mediator complex/CDK8 subcomplex. Phosphoacetylated H3 promotes transcriptional activation of many genes, including an IEG, EGR1. CDK8 knock-down results in decreased global levels of phosphoacetylated H3 [112]. Beside its role in transcription activation, CDK8 affects transcription elongation of IEGs. CDK8 depletion leads to reduced phosphorylation of Ser2 and Ser5 of RNA pol II CTD leading to reduced transcriptional elongation pace at these loci. Moreover, CDK8 depletion affects formation of a functional elongation complex (super elongation complex, SEC) formed by CDK7, CDK9, P-TEFb and BRD4 [111].

Kinase activity of CDK8 towards H3S10 is regulated by specific class of lncRNAs, called ncRNA-activating (ncRNAa). Long ncRNAas act in *cis* bringing enhancer and promoter region via Mediator complex to promote assembly of transcription factors and RNA pol II on the target mRNA promoter sites. Mediator with CDK8 phosphorylates H3S10 to activate transcription. Knocking down a given ncRNAa resulted in a specific decrease in H3S10 phosphorylation at its target gene [113]. This long-distance enhancer-promoter loops mediated by CDK8 might also contribute to transcriptional memory, where stimuli-induced genes show rapid transcriptional responses upon re-introduction of the stimulus. In consistence with that, CDK8 preferentially associated with the promoters of genes that show transcriptional memory after interferon-γ treatment [114]. Similar long-distance promoter-enhancer interactions exist also in super-enhancers (SEs), thus it is not surprising that CDK8 has a role in the regulation of SE-dependent genes. SEs are large clusters of enhancers loaded with particularly high levels of Mediator complex and transcription factors, regulating expression of genes crucial for cell identity and disease, including multiple oncogenes [115–117]. Best studied example for CDK8-SE connection was described in acute myeloid leukemia. Surprisingly, in those cells CDK8 restrains activation of SE-genes needed for normal hematopoiesis and cell differentiation, and inhibition of Mediator kinases CDK8 and 19 by cortistatin A (CA) has a therapeutic effect [118]. These results put an emphasis on the context-dependent action of CDK8, that can act both as an activator and repressor of gene expression.

Modulation of histone marks/chromatin is not the only mechanism by which CDK8 can regulate transcription. It can do so directly via phosphorylation of many transcription factors, including, among others, E2F1, NOTCH, p53, SMADs and STATs*.* In addition, CDK8 can modulate transcription in a very precise, signal-specific fashion. For example, after strong p21 activation, CDK8-mediator complex is selectively recruited to p53-target genes, augmenting p53 transcriptional program [111]. Similarly, in colon cancer cells, CDK8 protects β-catenin/T-cell factor (TCF)-dependent transcription from inhibition by E2F1 [119–121]. In addition, CDK8 is also involved in the transcriptional regulation of hypoxia-inducible genes. Hypoxia-inducible genes are pre-prepared for rapid transcription, carrying active, but paused, RNA pol II. With reduced oxygen level, hypoxia-inducible factor 1 alpha (HIF1A) recruits CDK8-bearing Mediator complex and SEC, what leads to increased phosphorylation of RNA pol II CTD and transcriptional elongation. Although HIF1A alone is able to bind to chromatin and induce histone acetylation, CDK8 is indispensable for the recruitment of transcriptional machinery and elongation progression [122]. Likewise, CDK8 enables transcriptional elongation by phosphorylation of RNA pol II at Ser2 in response to estrogen stimulation in breast cancer cells [123].

### Pim-1 proto-oncogene, serine/threonine kinase (PIM1)

PIM1 is another interphase H3S10 kinase with a documented role in cancer initiation and progression. Interestingly, PIM1 overexpression alone is not sufficient to induce tumorigenesis with high penetrance. In marked contrast, simultaneous overexpression of PIM1 and MYC dramatically accelerates MYC-induced lymphomagenesis, leading to formation of aggressive lymphomas in utero or around birth [124–128]. After stimulation with growth factor, PIM1 forms a complex with the dimer of MYC with its interacting protein MAX (MYC-associated factor X). The entire complex is recruited to the genomic locus of MYC targets, including IEGs: *FOSL1 and ID2*. There, PIM1 catalyzes phosphorylation of H3S10 in the nucleosomes at MYC-binding site what enables transcriptional activation of those genes. MYC and PIM1 colocalize at the sites of active transcription, and together regulate 20% of the MYC-target genes. Consistent with this, PIM1 inhibition reduced cellular transformation potential of MYC [63].

### IκB kinase α (IKKα, CHUK) and inflammatory pathway-induced kinases

In addition to the role in IEGs expression, H3S10 phosphorylation is required for the proper transcriptional response to proinflammatory signals activating NFκB, such as lipopolysaccharides (LPS), CD40 and tumor necrosis factor (TNF) [129–131]. Although these stimuli operate through distinct receptors (toll-like receptors—TLRs, TNF-family receptors, e.g., TNFR) and utilize different signaling intermediates upstream of NFκB, they elicit rapid H3S10 phosphorylation of at least a subset of promoters of genes regulated by these signals. IKKα, activated downstream of TNFR, functions in the nucleus and interacts with CREBBP acetyltransferase [129, 131]. The IKKα-CREBBP complex is recruited to NFκB- responsive promoters by RelA and mediates H3S10 phosphorylation and subsequent H3K14 acetylation. Other stimuli, such as LPS and TLR4, trigger p38 activation, what subsequently leads to similar chromatin modifications [130]. Inhibition of p38 decreases H3S10 abundance at promoters of certain LPS-inducible promoters and dramatically attenuates transcriptional responses to this pro-inflammatory signal, indicating that H3S10ph is required for full transcriptional activation of these genes. However, as histone’s H3 serine 10 is not followed by a proline, it cannot be directly phosphorylated by MAPKs [105, 107, 130]. Consistent with this, p38 has not been shown to directly associate with chromatin and phosphorylate H3S10, indicating that a p38-regulated kinase must be responsible for these epigenetic modifications. In fact, other studies indicate that LPS-induced cytokine production requires p38-regulated MSK1 or RSK2—well-established H3S10 kinases [132]. Interestingly, MSK1/2 knockout mice do not present an overt phenotype when unchallenged [13, 133], but are sensitized to LPS-induced endotoxic shock, indicating that MSKs exhibit anti-inflammatory functions [134]. Indeed, after LPS stimulation MSK1/2 knockout resulted in elevated levels of TNF, IL-6, and IL-12 production, but decreased production of the anti-inflammatory cytokine IL-10 [134, 135]. These data indicate that in vivo, MSK1 activity (and, possibly H3S10) downstream of TLR4, is a sort of a “safety fuse”, limiting LPS-induced inflammation. Similar mechanism of IL-10 secretion upon TLR4 activation has been shown for macrophages and B-cells, albeit different kinases are mediating these anti-inflammatory responses: macrophages utilize MSK1, whereas B-cells depend on RSK2 [136].

Given the importance of H3S10ph in mediating/maintaining tumor cell phenotype, oncogene transcription, inflammatory signaling pathways, cancer-related inflammation and immune escape, modulation of the deposition of this mark has a broad therapeutic potential. In addition, since kinases capable of H3S10 phosphorylation are able to regulate a wide range of substrates beyond H3S10, their inhibition has broad, pleiotropic effects. This pleiotropy makes them very attractive therapeutic targets, promising multidirectional and multi-layer activity. In fact, many of the enzymes catalyzing phosphorylation of H3S10 have long been considered targets for cancer therapy [88, 137–141]. However, the pleiotropy can also become a double edge sword, where inactivation of these multifunctional kinases results in unacceptable toxicities and, perhaps, heritable changes in chromatin architecture. In particular, the complex interplay of H3S10 phosphorylation with more stable modifications can actually have negative, long-lasting effects, and may thus present a danger in the clinical setting. Protein kinase inhibitors with proven effect on H3S10ph mark deposition and its cellular abundance, approved by FDA for clinical trials are listed in Table 2.

## Conclusions and perspectives

**Fig. 2:**
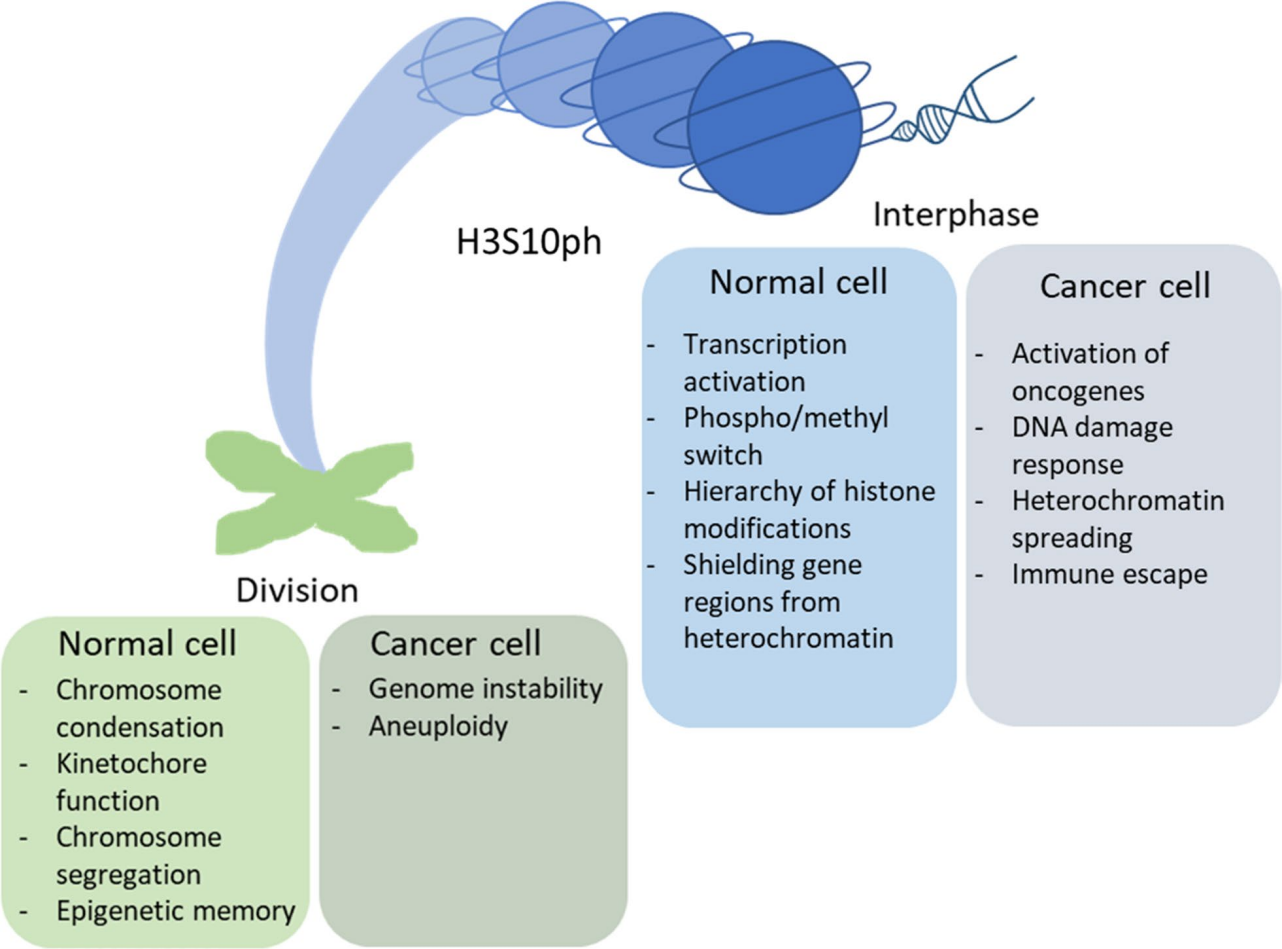
Effect of H3S10 phosphorylation on chromatin activity in normal and cancer cells. During interphase, enhancers-associated H3S10ph is involved in transcription activation and histone acetylation. By a ‘phospho-methyl switch’ it modulates epigenetic information coming from methylation of neighboring K9—by affecting H3K9me deposition it shields gene regions from heterochromatin. It also dictates spatiotemporal hierarchy of histone modifications. Deregulation of H3S10ph deposition can lead to cellular transformation via activation of protooncogenes, inhibition of developmental genes by heterochromatin spreading, genome instability caused by abnormal R-loops, and H3S10ph-mediated deregulation of inflammatory cytokines, potentially facilitating immune escape. During cell division H3S10ph is needed for proper chromosome condensation, kinetochore function and chromosome segregation. Deregulation of the deposition of this mark causes genome instability and aneuploidy caused by chromosome lagging or abnormal cytokinesis

Phosphorylation of Serine 10 on histone H3 is a dynamic histone posttranslational modification and an important epigenetic event that affects chromatin activity during numerous biological processes. It is responsible for the hierarchy of histone modifications involved in the formation of different protein binding platforms, inducing transcriptional activation by promoting histone acetylation, blockade of repressive epigenetic information and shielding gene regions from heterochromatin spreading. During cell division, this mark is essential for chromosome condensation and segregation. It is also involved in the function of specific DNA-RNA hybrids, called R-loops (Fig. 2).

Deregulation of H3S10ph seems to have a major effect on the cellular transformation process. Many of the enzymes catalyzing H3S10 phosphorylation and dephosphorylation are long-known oncogenic factors. Traditionally, they were thought to affect gene expression through the modulation of signaling pathways and the activity of transcription factors. Now, with more reports on the role of H3S10ph in tumor cells, it becomes clear that their aberrant activity can lead to more profound changes in the chromatin structure. An important aspect of the epigenetic regulation is its potential long-lasting effect, that, through the mechanism of epigenetic memory, can be transmitted during mitosis. It ensures long-term maintenance of the results of the activity of those enzymes, even after initial developmental and environmental stimuli have been removed. Thus, surveying chromatin changes should be incorporated during development of therapeutic compounds aiming at inhibition of those enzymes.

Given the broad substrate spectra of these kinases, the role of alterations in H3S10ph-associated chromatin structure/function triggered by their inhibitors is likely responsible for only a part of their anti-tumor activity; importantly, this part has not been broadly addressed and remains largely unknown. As the H3S10-regulated transcriptional activity is generally confined to specific gene sets, deconvolution of transcriptional program(s) disturbed by a given H3S10 kinase inhibitor would greatly improve understanding of their mechanism of action, and—more importantly—guide rational combinations with other molecularly directed drugs, immunotherapeutics, or conventional chemotherapy. Discovery of other links between chromatin remodeling and signaling pathways will help develop more precise, knowledge-driven therapeutic strategies.

## Data Availability

Not applicable.

## Abbreviations

Akt1: Protein kinase B
AURK: Aurora kinase
BRD4: Bromodomain-containing protein 4
CA: Cortistatin A
CDK8: Cyclin-dependent kinase 8
CLS: Coffin–Lowry syndrome
CTD: Carboxy terminal domain
EGF: Epidermal growth factor
H3S10ph: Phosphorylation of serine 10 on histone H3
HP1: Heterochromatin protein 1
IEGs: Immediate early genes
IKKα: IκB kinase α
JNK: C-Jun NH(2)-terminal kinase
LPS: Lipopolysaccharide
MAPK: Mitogen-activated protein kinase
MAP3K8: Mitogen-activated protein kinase kinase kinase 8
MAX: MYC-associated factor X
MSK1/2: Mitogen- and stress-activated kinases 1 and 2
NFκB: Nuclear factor kappa-light-chain-enhancer of activated B cells
PIM: Proto-oncogene serine/threonine-protein kinase
PKC: Protein kinase C
PP1: Protein phosphatase 1
P-TEF: Positive transcription elongation factor
PTM: Post-translational modification
RSK2: Ribosomal S6 kinase 2
SEs: Super-enhancers
SEC: Super elongation complex
TCF: T-cell factor
TFF1: Trefoil factor 1
TLR: Toll-like receptor
TNF: Tumor necrosis factor
TNFR: Tumor necrosis factor-family receptor
TPA: 12-O-tetradecanoylphorbol-13-acetate
